# Hospitalised patients with suspected 2009 H1N1 Influenza A in a hospital in Norway, July - December 2009

**DOI:** 10.1186/1471-2334-11-75

**Published:** 2011-03-24

**Authors:** Bjorn J Brandsaeter, Magnus Pillgram, Dag Berild, Harald Kjekshus, Anne-Marte B Kran, Bente M Bergersen

**Affiliations:** 1 Oslo University Hospital Aker, Department of Infectious Diseases, Trondheimsveien 235, 0514 Oslo, Norway; 2 Oslo University Hospital Aker, Department of Cardiology, Trondheimsveien 235, 0514 Oslo, Norway; 3 Oslo University Hospital Ulleval, Department of Microbiology, Kirkeveien 166, 0407 Oslo, Norway

**Keywords:** Influenza A H1N1, pandemic, hypertension, isolation, outcome

## Abstract

**Background:**

The main objective of this study was to describe the patients who were hospitalised at Oslo University Hospital Aker during the first wave of pandemic Influenza A (H1N1) in Norway.

**Methods:**

Clinical data on all patients hospitalised with influenza-like illness from July to the end of November 2009 were collected prospectively. Patients with confirmed H1N1 Influenza A were compared to patients with negative H1N1 tests.

**Results:**

182 patients were hospitalised with suspected H1N1 Influenza A and 64 (35%) tested positive. Seventeen patients with positive tests (27%) were admitted to an intensive care unit and four patients died (6%). The H1N1 positive patients were younger, consisted of a higher proportion of non-ethnic Norwegians, had a higher heart rate on admission, and fewer had pre-existing hypertension, compared to the H1N1 negative patients. However, hypertension was the only medical condition that was significantly associated with a more serious outcome defined as ICU admission or death, with a univariate odds ratio of the composite endpoint in H1N1 positive and negative patients of 6.1 (95% CI 1.3-29.3) and 3.2 (95% CI 1.2-8.7), respectively. Chest radiography revealed pneumonia in 24/59 H1N1 positive patients. 63 of 64 H1N1 positive patients received oseltamivir.

**Conclusions:**

The extra burden of hospitalisations was relatively small and we managed to admit all the patients with suspected H1N1 influenza without opening new pandemic isolation wards. The morbidity and mortality were similar to reports from comparable countries. Established hypertension was associated with more severe morbidity and patients with hypertension should be considered candidates for vaccination programs in future pandemics.

## Background

After the first cases of the 2009 H1N1 Influenza A were confirmed during week 30 in 2009, The Norwegian Ministry of Health asked the Norwegian hospitals and primary care facilities to activate their plans to cope with an expected massive wave of Influenza A patients [1-6]. On June 11, 2009, the World Health Organization raised the pandemic alert level to 6 [7,8]. Early observations indicated that up to 95% of infected patients were under the age of 50 years [9].

Concerns were raised that our health care facilities were not equipped or prepared to admit all the expected patients. The need for isolates, ventilators, antivirals and antibiotics were evaluated, and we feared that lack of equipment and personnel would cause extra strain on health care institutions. We also feared that secondary cases among the personnel would further increase the workload and compromise care.

The aim of the study was to describe underlying medical conditions, clinical features, and outcome of the first 2009 H1N1 Influenza A patients at Oslo University Hospital Aker (OUHA) and to compare these patients with the patients admitted with suspected 2009 H1N1 Influenza A, but who tested negative for Influenza A. We also investigated secondary cases and the extra workload the pandemic put on our hospital facilities and employees.

## Methods

### Oslo University Hospital Aker (OUHA)

OUHA serves an area of 180 000 inhabitants. 73 000 of these live in two different districts of Oslo with approximately 40% non-ethnic Norwegians. The rest live in sparsely populated communities, suburbs and rural areas outside Oslo with about 2-3% non-ethnic Norwegians. At the time of the pandemic the Department of Medicine had 129 beds. The Department of Infectious Diseases shared 16 beds divided equally with the Department of Gastroenterology. The Intensive Care Unit (ICU) consisted of two parts: six general intensive care beds without the possibility of ventilator but with equipment for continuous positive airway pressure (CPAP) and bilevel positive airway pressure (BiPAP) treatment. The other part of the ICU had six beds with the possibility for ventilator treatment. There were no paediatric, gynaecologic or obstetric departments at the hospital.

### Isolation facilities and hygienic precautions

As a preparation for the pandemic all 16 beds were made available for the Department of Infectious Diseases if needed. There were six single rooms and five double rooms. The hospital had no negative air-pressure isolates and few patient rooms with antechamber and separate bathrooms. In June 2009, all staff in the involved departments completed an e-learning program about hygienic rules for handling influenza patients. At the end of October 2009 all employees at the Emergency, Intensive Care and Infectious Disease Departments were offered vaccination against Influenza A. In addition to disposable gowns and gloves, the hospital staff used surgical masks for protection. If vaporizing procedures were performed the staff used health care respirator masks. All suspected influenza patients admitted to the hospital were initially isolated in single rooms in the Emergency Department. The patients were quickly evaluated by nurse and physician and then moved to either the Intensive Care Unit or to the Department of Infectious diseases. There were no strict criteria for ICU admission, but in general patients presenting with several positive SIRS criteria such as heart rate >100, respiratory rate >25, and fever > 38°C and hypoxia, were referred to the ICU.

### Study Design

From 15 July to 30 November 2009, all hospitalised patients above 18 years of age with suspected H1N1 Influenza A were enrolled in the study. The responsible physician registered clinical signs and symptoms, underlying medical conditions, bacterial findings, selected laboratory tests, treatment and outcome for all patients in a standardized form. Thus, registration of pre-existing medical conditions was based on patient history on admission and available pre-existing data in the hospital electronic patient registry. Patients with suspected influenza were all coded with the ICD-10 code Z11.5 in our electronic patient journal system. If pandemic H1N1 was confirmed, the patient received an additional ICD-10 code (J09). This enabled us to track and record both the suspected and confirmed cases. The inclusion of new patients ended 30 November 2009, but patients were followed until 15 December 2009.

### Tests

All patients with suspected H1N1 Influenza A were tested for virus using nasopharyngeal- and throat-swabs. All specimens were analysed with RT-PCR. In brief, purification of viral RNA from respiratory specimens was performed using the bioMérieux easyMAG Nuclisense extractor (bioMerieux, Marcy-l'Etoile, France) according to the manufacturer's instructions. RT-PCR was performed with One-Step RT-PCR kit (Qiagen, Hilden, Germany), using primers and probes to detect Influenza A virus and 2009 pandemic H1N1 Influenza A virus subtype as described elsewhere [10,11]. During the peak of the pandemic outbreak in October 2009, samples positive in the influenza A test were not tested for subtype, but all influenza A were considered to be pandemic H1N1. During the study period, specific tests for pandemic H1N1 influenza A were performed on 54% of all specimens analyzed for influenza in Norway, and 99.9% of the sub typed influenza viruses were confirmed to be of pandemic H1N1 subtype.

### Definition

We defined the suspicion of influenza as a patient with fever and/or respiratory symptoms and/or generalized symptoms of infection, such as myalgia, head-ache and chills. We defined fever as a temperature > 38°C. We used Kendall GENIUS 2 Infrared Tympanic Electronic Thermometers and nurses who had received specific training in operating the tympanic thermometers conducted temperature measurements in this study.

### Treatment

Oseltamivir was the preferred antiviral drug. Zanamivir was available from October 2009, primarily used for prophylaxis in pregnant women.

Hospital antibiotic guidelines recommend that community acquired pneumonias are treated with crystalline penicillin in monotherapy. Normally a combination of penicillin and gentamicin is used for septic patients, however, after experiencing one case of acute severe renal failure in H1N1 positive patients after gentamicin treatment, septic H1N1 patients with hypotension or oliguria were given cefotaxime in monotheraphy.

### Ethics Statement

The hospital's Data Protection Officer approved the study protocol on behalf of the regional ethics committee. The research involved no risk to subjects nor involved any procedures for which written consent is required.

### Statistical analysis

Statistical analyses were performed with SPSS 15.0 (Chicago, Illinois, US). Categorical data were analysed using the Fisher exact test. Data for continuous variables are reported as median (range) and for categorical variables as percentages. Logistic regression was used in the univariate and multiple regression analyses. A p-value of less than 0.05 was considered significant. The multivariate analyses were performed by entering the variables that had a p-value of < 0.1 with subsequent removal of the least significant variables in a stepwise fashion.

## Results

### Patients with confirmed influenza A (H1N1)

**Figure 1:**
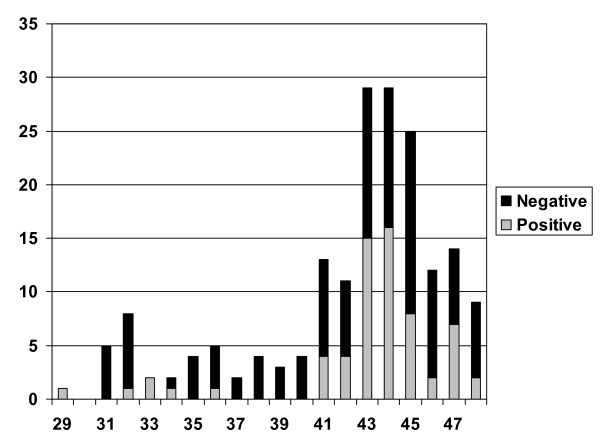
**Absolute number of positive and negative H1N1 tests according to week after the first positive test in week 29**. There was a marked increase from week 41 with a peak of patients in weeks 43 to 45.

From 15 July to 30 of November 2009 a total of 182 cases of suspected pandemic H1N1 were hospitalised, of which 64 (35%) were confirmed pandemic H1N1 with RT-PCR (Fig1). The Department of Gastroenterology and Infectious Diseases had 498 admissions in this given time-period in 2008 and 495 admissions in 2009.

The median age of patients with a positive influenza-test was 42 years (19 - 69 years), and there were 26 men (41%) and 38 women (59%). The median time from onset of illness to hospital admission was three days (range 0 - 20) and the median length of stay in hospital was three days (0.3 - 47).

Fifty five (86%) of the 64 patients who tested positive, had one or more underlying medical conditions. Patient characteristics are shown in Additional file 1. Chronic pulmonary disease was the most common predisposing risk factor seen in 34% of the patients with Influenza A (Additional file 1). Three patients (5%) were pregnant, of whom one had a chronic pulmonary disease.

### Demographics and vital signs at admission

The patients with positive H1N1 tests were significantly younger, had a higher heart rate on admission, and were more often non-ethnic Norwegians and healthcare workers, compared to the H1N1 negative patients. Established hypertension was more common in the H1N1 negative group (Additional file 1).

### Fever

Only 31 (48%) of the patients with confirmed pandemic Influenza A had fever on admission (temperature measurement available for 63 of 64 patients). Information on the pre-admission use of antipyretic drugs (paracetamol, ibuprofen and various immunosuppressants) was scarcely available (N = 25 patients).

### Bacteriology and antibiotics

Blood cultures were obtained in 51 patients (80%). There was growth of Streptococcus pneumonia in two cultures and of Streptococcus viridans in one, altogether in 6%. All strains were susceptible to penicillin. Six patients received antibiotics before admission. During the hospital stay 33 patients (52%) received 47 treatment-courses of antibiotics. Median duration of antibiotic treatment was six days The most common antibiotics used were penicillin and cefotaxime. Eight patiens received penicillin, where four of these changed to cefotaxime. Similarly, six patients received cefotaxime monotherapy, and 4 patients changed from cefotaxime to penicillin.

### Radiology

Chest radiography was performed in 59 (92%) of the patients with influenza A and revealed pneumonia in 24 patients (41%). The comparative figures for H1N1 negative patients were 101 x-rays performed (86%) and 45 diagnosed with pneumonia (45%).

### Antiviral treatment

One pregnant patient was offered treatment but refused. Seven patients (11%) started oseltamivir before admission to hospital, 44 (69%) started on the same day they were admitted, the remaining eight started oseltamivir one to four days after admission. No patients received zanamivir. Four patients died - of these one did not receive antiviral treatment, one started oseltamivir two days prior to hospital admission and two patients started at admission. Six patients needed mechanical ventilation - two of these had started oseltamivir before admission, the remaining four started at admission.

### Vaccination

Six of the 64 patients with confirmed H1N1 Influenza A had been vaccinated with 2009 H1N1-specific vaccines. Two of these received their vaccine the same day as they were admitted; the remaining four were vaccinated one, two, 11 and 21 days prior to admission. One of the six vaccinated patients died. This patient received the vaccine 21 days prior to admission. He had an aggressive chronic lymphatic leukaemia.

### Number of admitted patients and length of stay

Figure 1 shows the total number of admissions (both patients with positive and negative tests) according to week. There was a marked increase in the number of admissions between week 41-47 and especially between weeks 43-45. There was no recorded wave of 2009 pandemic Influenza A before this but there were some sporadic cases.

**Figure 2:**
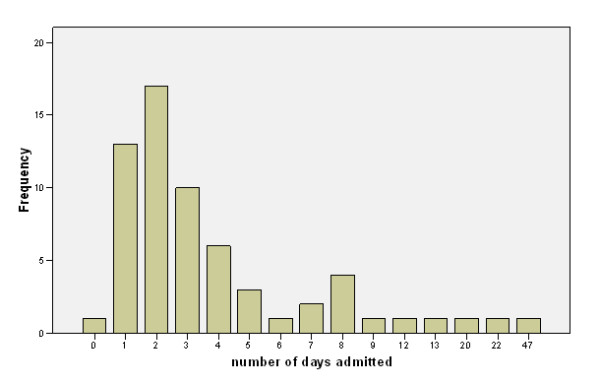
**Frequency of length of stay**.

The length of stay for the patients with a positive H1N1 test was relatively short (Figure 2) with a median stay of 3 days.

### ICU admissions and outcome

Of the patients with confirmed Influenza A, 27% were admitted to the ICU compared to 15% of H1N1 negative patients (ns). Clinical findings including fever, tachycardia, tachypnea, and hypoxia was more prevalent in the ICU admitted H1N1 positive patients compared to the H1N1 positive patients admitted to the Infectious Disease (ID) ward consistent with the selection criteria for ICU admission.

Putative associations between underlying medical conditions and demographics were explored by univariate analyses using admission to the ICU as the dependent variable, and are described in Additional file 2. The H1N1 positive patients admitted to the ICU tended to be younger, have a higher heart rate, and had a higher Body Mass Index (BMI) than the H1N1 negative ICU admitted patients.

Hypertension was the only medical condition that was significantly associated with a more serious outcome defined by ICU admission or death, with a univariate oddsratio of the composite endpoint in H1N1 positive and negative patients of 6.1 (95% CI 1.3 - 29.3) and 3.2 (95% CI 1.2 - 8.7), respectively (Additional file 2).

Possible associations were further explored in a multiple regression analysis with ICU admission as the dependant variable. In the H1N1 negative patients, hypertension did not remain significant in a stepwise model, nor did any of the other variables (diabetes, chronic heart failure and excessive alcohol consumption). However, in the H1N1 positive patients, hypertension remained significant with an OR of approximately 7 and p < 0,03 in all models.

Ten patients improved rapidly and left the ICU within one (six patients) or two days (four patients). Most of them were dehydrated and had moderate respiratory problems. One had Addisons disease and was admitted to the ICU because of an Addison crisis. Among the seven patients with three or more days in the ICU, two patients had severe pneumococcal sepsis. Both survived after 26 and 14 days in the ICU and they both needed mechanical ventilation. In total four patients (6%) died. All had at least one risk factor (one patient had one risk factor, two had two risk factors and one had three risk factors). One was multi-handicapped and ventilator was not indicated of ethical reasons. Another young patient with severe obesity (BMI 37) had severe respiratory problems on admission and died after six days of mechanical ventilation and four days with extracorporeal membrane oxygenation (ECMO). The patient's mother, who had rheumatoid arthritis treated with hydroxychloroquine sulphate, was admitted to our hospital after seven days with influenza symptoms. After two weeks in the ICU she died of a viral pneumonia. The last patient who died had an underlying aggressive malignant haematological disease.

### Secondary cases and absence from work

We had no secondary H1N1 Influenza A among the staff and no increase in employers being absent from work at the Departments of Infectious Diseases and Intensive Care. One nurse on another ward who had been in contact with one of the patients with influenza, tested positive for H1N1 Influenza A, but recovered within seven days.

### Patients with negative tests for Influenza A

**Figure 3:**
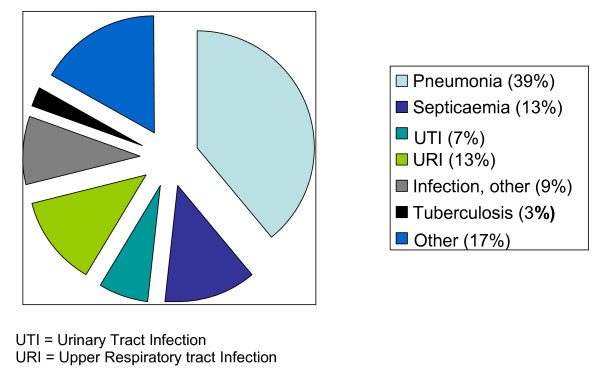
**Main diagnoses among the 118 patients with negative RT-PCR tests for Influenza A**.

The most common discharge-diagnoses for these patients were pneumonia, septicaemia and other infections (e.g. urinary tract, upper respiratory tract) (Figure 3). The remaining patients had non-infection diagnoses like myocardial infarction, trauma with fever and gastrointestinal symptoms. Eight of the influenza negative patients (7%) died. The characteristics of the patients with negative tests for Influenza A are given in Additional file 1. Signs and symptoms on admission could not help us determine who would have a positive test for Influenza A.

## Discussion

The most interesting findings in this study were: 1) pre-existing hypertension was associated with more severe morbidity and 2) the burden of patients with negative influenza tests and the logistical aspect of admitting a large number of possibly infected patients did not compromise care, nor did it create secondary infections in our staff despite relatively simple hospital facilities. The finding that two of the four patients who died were related should lead to further investigation into possible genetic factors associated with complicated outcomes.

Early in the course of the pandemic we realized that two thirds of the patients with suspected influenza had negative influenza tests. The H1N1 positive patients were younger, more often non-ethnic Norwegians, had a higher heart rate on admission and less hypertension compared to the H1N1 negative patients. However, the range of variation was large and no single factor or set of factors could reliably differentiate H1N1 positive from negative patients.

The number of patients that were admitted to an intensive care unit is in accordance with reports from other centres [12]. Four of seventeen (24%) patients in the ICU died which is somewhat higher than in a report from Australia and New Zealand [13].

Although there were more patients with established hypertension among the H1N1 negative patients, hypertension was found to be a predictor of a more serious outcome for both H1N1 positive and negative patients. However, only in the H1N1 positive patients remained hypertension a significant predictor in a multivariate analysis. The prevalence of hypertension in H1N1 positive patients with a more severe morbidity has not been widely reported in recent papers [14,15], however in an earlier report from Thailand describing risk factors for a fatal outcome in influenza patients (subtype A/H3N2, A/H1N1 and type B) they found an increased prevalence of hypertension in fatal cases of Influenza (age adjusted OR 4,7) supporting our findings [16].

We learned that clinical evaluation of the suspected influenza patients in the Emergency Room could not predict the result of an influenza test. But we could, to a certain extent, predict that patients with one or more co-morbidities would need intensive care treatment.

Few patients were seriously ill at the time of admission despite a low proportion of patients having started antiviral treatment or antibiotics in the days before. Blood cultures were obtained from 80% of the patients and were positive in only 6%. All strains were susceptible to penicillin. The most seriously ill patients that survived were two young men with pneumococcal sepsis that needed ventilatory support. They had no underlying medical conditions and in future planning for influenza pandemics pneumococcal vaccination should be discussed.

The deceased patient with aggressive chronic lymphatic leukaemia received the vaccine 21 days prior to admission, and it is doubtful whether the patient was able to produce a sufficient immunological response to the vaccine antigen.

The number of patients with pre-existing underlying medical conditions was higher than in many other reports. This may indicate that we tested too few patients. The patients that died all died of respiratory failure, but the number of deaths in this study is too low for meaningful statistical analysis.

The areas with a high percentage of non-ethnic Norwegians are also more densely populated, therefore our finding of a higher percentage of non-ethnic Norwegians with H1N1 positive tests could simply be due to a higher infection rate in densely populated areas. The finding that non-ethnic Norwegians tended to have a lower than average admission rate to the ICU (ns) does not support that a putative higher susceptibility to H1N1 infection among non-ethnic Norwegians is associated with a worse outcome. However, the numbers are small and this aspect warrants further study.

We may have missed to diagnose influenza in some of the patients: firstly, our criteria for testing may have excluded some influenza-patients from being tested. Secondly, some of the patients with signs of respiratory tract disease were probably infected with pandemic H1N1 despite negative tests. Nevertheless, 65% of the patients who were tested had negative tests for Influenza A. False negative results may occur if the amount of virus present in the collected samples is below the detection limit of the RT-PCR, either due to prolonged time from onset of symptoms to sample collection, limited local viral replication in nasopharyngeal epithelial cells or inadequate sample collection. The patients with negative H1N1 tests counted for twice as many patients as the patients with positive tests and contributed to a great burden to the hospital because most of them had to be isolated until their H1N1-status was known. In the planning of future pandemics the health care system must take into account the extra burden of all patients with suspected and not only proven disease.

During the peak of patient admissions we were forced to use some of the rooms without separate entrances. We therefore had to change to protective clothing in the corridor, which was not optimal. In addition we only used surgical mouth masks and not health care respirator masks (unless we performed vaporising procedures). In spite of this, we had no secondary cases of Influenza A at our ward or among our staff. This could be due to mass vaccination early in the pandemic. In general, vaccination of health care workers reduces the all-cause mortality of older patients by approximately 40% [17]. We evaluated on a daily basis if it was necessary to start using our planned cohort isolation ward, something we never had to do. The rapid daily services provided by the Department of Microbiology enabled us to clear the ward and make room for new patients at a high speed. During the peak of admissions we managed to place all our patients in the Department of Infectious Diseases and Intensive Care Unit except at one occasion where we had to isolate two patients in the Department of Cardiology.

## Conclusions

Despite suboptimal facilities, such as lack of negative air-pressure isolation rooms, we were capable of taking care of the large burden of patients by increasing our capacity from eight to 16 beds and by using the ICU. We did not see any secondary cases among our staff. The planned cohort-isolation unit was never in use. Quick and reliable service from the virus-laboratory with results of influenza testing the same day was necessary for a high turnover of patients.

Planning for future pandemics should take into account that some patients can acquire serious pneumococcal infections and that many patients with suspected H1N1 influenza have negative H1N1 tests. Hypertension was the only pre-existing medical condition that was associated with a more severe morbidity and outcome in H1N1 positive patients. Thus, patients with hypertension should be considered candidates for H1N1 vaccination programs in future pandemics.

## Competing interests

The authors declare that they have no competing interests.

## Authors' contributions

BJB co-conceived the study, participated in data collection, statistical analysis/interpretation and wrote the majority of the manuscript. MP contributed to the study design, data collection and statistical analysis/interpretation and wrote parts of the manuscript. DB contributed to the study design, participated in data collection and wrote parts of the manuscript. HK contributed to the concept and design of the study, data collection and statistical analysis/interpretation and wrote parts of the manuscript. AMBK contributed to the section on RT-PCR and microbiology. BMB conceived the study, participated in data collection and supervised the project. All authors have contributed to the revision of the manuscript.

## Pre-publication history

The pre-publication history for this paper can be accessed here:

http://www.biomedcentral.com/1471-2334/11/75/prepub

## Supplementary Material

Additional file 1 **Table 1**. Characteristics and outcome of all included patients. Click here for file

Additional file 2 **Table 2**. Comparison of patient characteristics with respect to either ID Ward or ICU admission and odds-ratio for ICU admission versus ID Ward admission given certain patient characteristics. Click here for file
