# Supplementary material for: Hospitalised patients with suspected 2009 H1N1 Influenza A in a hospital in Norway, July - December 2009
Source: BMC Infect Dis. 2011 Mar 24;11:75. doi: 10.1186/1471-2334-11-75 (PMC3078866; doi:10.1186/1471-2334-11-75)
Supplement: Additional file 1 — Table 1. Characteristics and outcome of all included patients. [file 1471-2334-11-75-S1.DOC]

|  | **H1N1 Pos**  **N = 64** | **H1N1 Neg**  **N = 118** | **p-**  **value** |
| --- | --- | --- | --- |
| **Demographics:** | | | |
| Age | 42 (19-69) | 57 (17 - 89) | p=0,002 |
| Female gender | 59,4 % | 55,1 % | ns |
| Non-ethnic Norwegian | 42,2 % | 20,3 % | p=0,002 |
| Health care worker | 7,8 % | 1,8 % § | p=0,046 |
| Current smoker | 28,1 % | 24,1 % | ns |
| Excessive alcohol consumption | 10,9 % | 6,9 % | ns |
| **Laboratory findings on admission:** | | | |
| CRP (mg/l) | 35 (0 – 338) | 84 (0 - 429) | p=0,005 |
| Leukocyte count (*10^9^/l) | 7,4 (1,8 – 127) | 10,0 (0,7 – 47,6) | p<0,001 |
| Pneumonia seen on X-ray | 40,7% § | 44,6 % * | ns |
| **Clinical status on admission:** | | | |
| Temperature ≥ 38° C | 49,2 % § | 44,2 § | ns |
| Systolic BP ≤ 100 mmHg | 7,8 % | 7,0 % § | ns |
| Heart rate ≥ 100 beats/minute | 59,4 % | 41,6 % § | p=0,023 |
| Respiratory rate ≥ 25 | 42,9 % ¤ | 36,5 % * | ns |
| SpO2 ≤ 90% | 28,3 % § | 19,0 % * | ns |
| **Pre-existing co-morbidies:** | | | |
| Diabetes | 17,2 % | 17,8 % | ns |
| Chronic lung disease | 34,4 % | 32,2 % | ns |
| Chronic heart failure | 15,6 % | 21,2 % | ns |
| Chronic kidney failure | 3,1 % | 7,6 % | ns |
| Chronic liver failure | 4,7 % | 5,1 % | ns |
| Neurological disease | 17,2 % | 11,0 % | ns |
| Autoimmune disease | 12,5 % | 12,7 % | ns |
| Hypertension | 12,5 % | 28,2 % | p=0,016 |
| Immune deficiency/suppresion | 9,4 % | 9,4 % | ns |
| Obesity | 20,3 % | 23,9 % * | ns |
| Pregnancy | 4,7 % | 1,7% | ns |
| **Outcome:** | | | |
| In-hospital mortality | 6,2 % | 8,2 % | ns |
| Admission to ICU | 26,6 % | 15,3 % | ns |

§ denote 1-5 missing values. # denote 6-10 missing values. ¤ denote 11-20 missing values. * denote >20 missing values. Continuous variables are reported as median (range) and categorical variables as percentages.
