# Supplementary material for: Hospitalised patients with suspected 2009 H1N1 Influenza A in a hospital in Norway, July - December 2009
Source: BMC Infect Dis. 2011 Mar 24;11:75. doi: 10.1186/1471-2334-11-75 (PMC3078866; doi:10.1186/1471-2334-11-75)
Supplement: Additional file 2 — Table 2. Comparison of patient characteristics with respect to either ID Ward or ICU admission and odds-ratio for ICU admission versus ID Ward admission given certain patient characteristics. [file 1471-2334-11-75-S2.DOC]

|  | **H1N1 Pos** | | | **H1N1 Neg** | | |
| --- | --- | --- | --- | --- | --- | --- |
|  | ICU admitted N=17 | ID ward N=47 | p-value/  OR (95% CI) | ICU admitted N=18 | ID ward N=100 | p-value/  OR (95% CI) |
| Age | 41 (19-69) | 42 (19-68) | ns | 69,5 (24-89) | 55 (17-89) | ns |
| Female gender | 9/17 (52,9 %) | 29/47 (61,7 %) | 0,7 (0,2-2,1) | 8/18 (44,4 %) | 57/100 (57,0 %) | 0,6 (0,2-2,1) |
| Non-ethnic Norwegian | 5/17 (29,4 %) | 22/47 (46,8 %) | 0,5 (0,1-1,6) | 2/18 (11,1 %) | 22/100 (22,0 %) | 0,4 (0,1-2,1) |
| Current smoker | 6/17 (35,3 %) | 12/47 (25,5 %) | 1,6 (0,5-5,2) | 6/17 (35,3%) | 20/91 (22,0 %) | 1,9 (0,6-5,9) |
| Excessive alcohol consumption | 3/17 (17,6 %) | 4/47 (8,5 %) | 2,3 (0,5-11,6) | 3/18 (16,7 %) | 5/98 (5,1 %) | 3,7 (0,8-17,2) |
| CRP (mg/l) | 102 (18-304) | 30 (0,4-338) | P<0,001 | 86,5 (4-429) | 83,5 (0,4-380) | ns |
| Leukocyte count (*10^9^/l) | 6,8 (2,0-127,0) | 7,7 (1,8-20,1) | ns | 8,9 (0,7-33,0) | 10,4 (3,5-47,6) | ns |
| Pneumonia seen on X-ray | 10/17 (588 %) | 14/42 (33,3 %) | 2,9 (0,9-9,1) | 9/15 (60,0 %) | 36/86 (41,9 %) | 2,1 (0,7-6,4) |
| Temperature ≥ 38° C | 12/17 (70,6 %) | 19/47 (41,3 %) | 3,4 (1,0-11,3) § | 8/18 (44,4 %) | 42/97 (44,2 %) | 1,0 (0,4-2,8) |
| Systolic BP ≤ 100 mmHg | 1/17 (5,9 %) | 4/47 (8,5 %) | 0,7 (0,1-6,4) | 3/18 (16,7 %) | 5/97 (5,2 %) | 3,7 (0,8-17,0) |
| Heart rate ≥ 100 beats/minute | 14/17 (82,4 %) | 24/47 (51,1 %) | 4,5 (1,1-17,6) § | 8/18 (44,4 %) | 39/95 (41,1 %) | 1,1 (0,4-3,2) |
| Respiratory rate ≥ 25 | 9/12 (75,0%) | 12/37 (32,4 %) | 6,3 (1,4-27,4) § | 9/14 (64,3 %) | 22/71 (31,0 %) | 4,0 (1,2-13,4) § |
| SpO2 ≤ 90% | 10/15 (66,7 %) | 7/45 (15,6 %) | 10,9 (2,8-41,6) § | 5/11 (45,5 %) | 11/73 (15,1 %) | 4,7 (1,2-18,1) § |
| Diabetes | 3/17 (17,5 %) | 8/47 (17,0 %) | 1,0 (0,2-4,5) | 6/18 (33,3 %) | 15/100 (15,0 %) | 2,8 (0,9-8,7) |
| Chronic lung disease | 7/17 (41,2 %) | 15/47 (31,9 %) | 1,5 (0,5-4,7) | 8/18 (44,4 %) | 30/100 (30 %) | 1,9 (0,7-5,2) |
| Chronic heart failure | 4/17 (23,5 %) | 6/47 (12,8 %) | 2,1 (0,5-8,6) | 7/18 (38,9 %) | 18/100 (18 %) | 2,9 (1,0-8,5) |
| Chronic kidney failure | 0/17 (0,0 %) | 2/47 (4,3 %) | 0,0 (0,0) | 2/18 (11,1 %) | 7/100 (7,0 %) | 1,7 (0,3-8,7) |
| Chronic liver failure | 3/17 (17,6 %) | 0/47 (0,0%) | 0,1 (na) | 0/18 (0,0%) | 6/100 (6,0 %) | 0,0 (na) |
| Neurological disease | 5/17 (29,4 %) | 6/47 (12,8 %) | 2,8 (0,7-11,0) | 4/18 (22,2 %) | 9/100 (9,0 %) | 2,9 (0,8-10,7) |
| Autoimmune disease | 3/17 (17,6 %) | 5/47 (10,6 %) | 1,8 (0,4-8,5) | 0/18 (0,0 %) | 15/100 (15,0 %) | 0,0 (na) |
| Hypertension | 8/17 (29,4 %) | 3/47 (6,4 %) | 6,1 (1,3-29,3) § | 9/18 (50,0 %) | 24/99 (24,2 %) | 3,1 (1,1-8,8) § |
| Immune def/suppr | 2/17 (11,8 %) | 4/47 (8,5 %) | 1,4 (0,2-8,6) | 2/18 (11,1%) | 9/100 (9,1 %) | 1,3 (0,3-6,3) |
| Obesity | 5/17 (29,4 %) | 8/47 (17,0%) | 2,0 (0,6-7,4) | 1/7 (14,2 %) | 15/60 (25,0 %) | 0,5 (0,1-4,5) |
| In-hospital mortality | 4/17 (23,5 %) | 0/47 (0,0 %) | 0,0 (na) | 6/18 (33,3 %) | 2/100 (2,0 %) | 21,0 (3,8-118,8) |

§ denote p<0,05
